# Major incident in Kent: a case report

**DOI:** 10.1186/s13049-015-0152-9

**Published:** 2015-09-22

**Authors:** Sophie Elizabeth Jap Hardy

**Affiliations:** The Medway Maritime Hospital, Kent, England

## Abstract

A major incident was declared after a road traffic accident involving 150 cars and 200 people in Kent, England. The emergency services oversaw coordination of the scene, recovery and triage of casualties and transfer of patients to hospital. The crash was one of the worst seen on British roads and it has been hailed as a miracle that there were no deaths and very few serious injuries.

This case report is a retrospective analysis of the regional health system’s response to the crash. The structure is based on the content of a report submitted using an online open access template for major incident reporting (Scand J Trauma Resusc Emerg Med 22: 5, 2014; http://www.majorincidentreporting.org). A more comprehensive analysis of the incident has also been the theme of a Masters thesis (Hardy S. Reporting Major Incidents in England: Putting Theory into Practice. England: Queen Mary’s University of London; 2014).

## Introduction

On 5th September 2013, a road traffic accident occurred on the Sheppey Crossing bridge in Kent. It happened at 07:15 under thick fog where visibility was reduced to 25 yards. As the fog lifted, it was evident that the pile up involved cars extending across most of the 1270 metre long bridge. There were 69 casualties but no fatalities occurred. Although declared a major incident, there was minimal disruption to the routine emergency and healthcare services.

### Pre-incident data

**Fig. 1:**
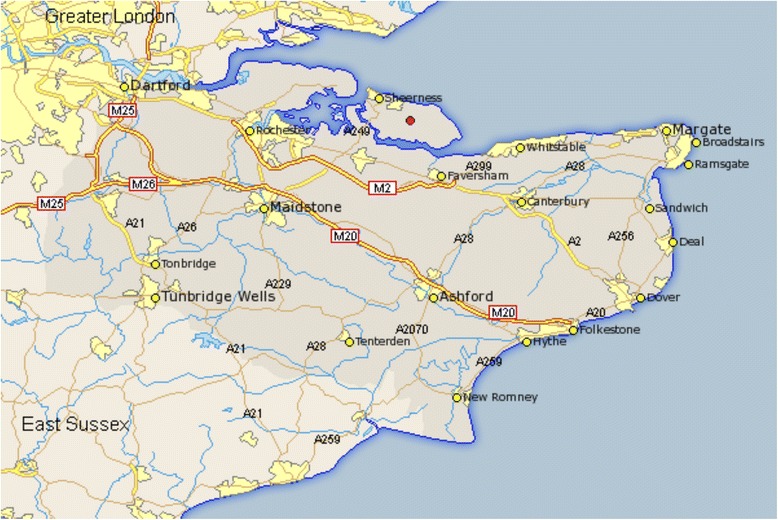
The Isle of Sheppey with the Sheppey Crossing bridge carrying the A249

The Isle of Sheppey is an island in the district of Swale off the North coast of Kent in England (Fig. 1). It has an area of 36 square miles and a population of 37,852. The population density of Swale is 3.49 people per hectare. Many of the inhabitants commute over one of two bridges that connect the Isle of Sheppey to mainland Kent. The Kingsferry Bridge is a combined single carriageway road and railway bridge and was the only connection to the mainland until the Sheppey crossing bridge was built in 2006. The Sheppey crossing runs alongside the Kingsferry Bridge and carries the A249 trunk road. It has 4 lanes with a 70mph speed limit and does not have a hard shoulder. It is unlit and has no matrix warning signs. Telecommunications are a vital part of England’s infrastructure and are managed by competitive, commercial companies. As category 2 responders, telecommunications “fixed” and “mobile” network providers can be called upon for cooperation during major incidents.

### Emergency Medical System (EMS) background

**Fig. 2:**
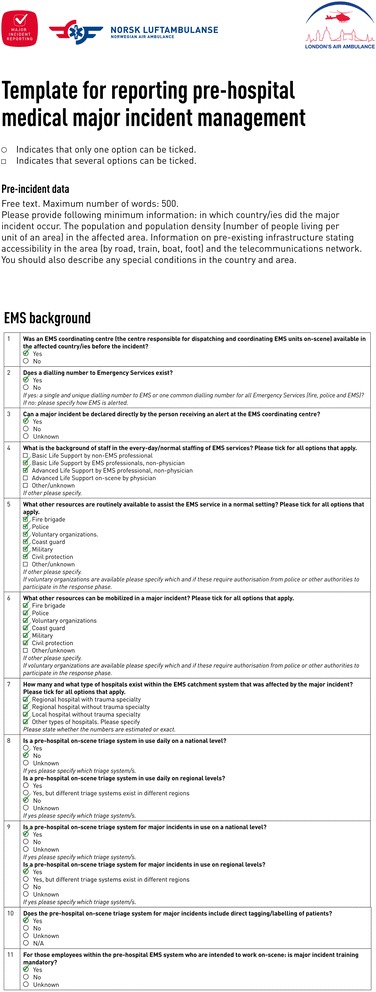
Extract from the template by Fattah et al. [1, 6] giving details of Emergency Medical Services (EMS) background

Emergency medical response in the UK is provided by local ambulance services known as National Health Service (NHS) trusts. The South East Coast Ambulance (SECAmb) service NHS trust covers Brighten & Hove, East Sussex, West Sussex, Kent, Surrey and North east Hampshire, an area of 3600 square miles. Emergency (999) calls are put through to one of 3 Emergency Operations Centres (EOCs). From here they are triaged to provide an appropriate response. As soon as initial reports indicate that a major incident may have occurred, the senior person on duty at the EOC will initiate the major incident plan. Figure 2 shows EMS background data from the online report [1].

### Incident characteristics

**Fig. 3:**
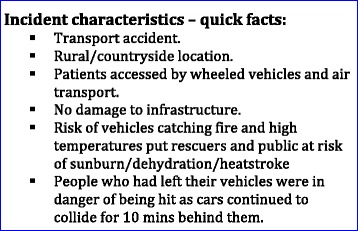
Incident characteristics overview

Despite heavy fog and low visibility some drivers approached the Sheppey bridge at speed and without lights. This culminated in a pile up involving over 150 cars. The crash continued for 10 min as vehicles continued to hit each other. More than 200 people were assessed at the scene and 37 of these required hospital treatment. A further 32 people presented to minor injuries units. The Bridge was closed for 10 h following the incident to allow for assessment and treatment of casualties and for a full investigation and clear up operation to be carried out. As the fog lifted, the 30 °C heat and the prolonged period stranded outside began to put those remaining on scene at risk from dehydration, lack of food and exacerbation of pre-existing illnesses (Fig 3).

### EMS response data

**Fig. 4:**
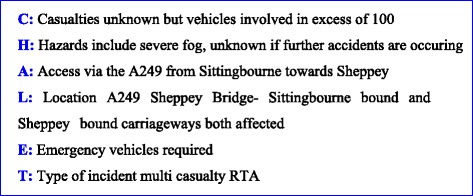
The CHALET mnemonic is used to report a major incident

The first ambulance responders on scene reported that hundreds of cars were involved with some priority 1 (P1) casualties (Fig 4). The incident response lasted for just over 10 h (Table 1) and the Sheppey Crossing was re-opened to routine traffic at 17:30 that day.

**Table 1:** Major incident timeline

Time	Action
07:15	Incident occurs
07:17	Initial call to emergency services from a member of the public: incident involving 3–5 vehicles on the southbound carriageway of the Sheppey bridge
07:22	Several calls received: approximately 50 vehicles involved
07:25	Kent police arrive on scene
07:26	First ambulance arrives on scene
07:30	Silver officer on-call hears news of large Road Traffic Accident on Sheppey Bridge but too far away from scene. A second silver officer is appointed to go on scene while the on-call silver officer goes to the nearest EOC
07:34	Kent Fire services arrive on scene
07:35	SECAmb manager to attend the scene arrives and assumes the role of AIC until the silver officer arrives at 10:30
07:40	First AIC meets with managers from police and fire services
07:54	Second manager arrives on scene and assists first AIC in formulating a plan of initial action
08:15	The first AIC declares a Major Incident and reports her assessment of the scene to the EOC using CHALET acronym (see box 2). She also requests more managers. Several ambulances are deployed.
08:17	Casualty Clearing Station established
08:24	MMH informed of Major Incident
08:30	Air ambulance team arrives and directed to the most severely injured patient. This patient is not deemed unwell enough to require helicopter transfer so the air ambulance team remain on scene to assist with other casualties.
08:41	HALO deployed to MMH
09:00	MMH declares a Major Incident
	First patient evacuated from scene.
	A large number of “walking wounded” are the first to arrive at the nearest Hospital, MMH
09:02	8 P2 patients identified, 2 of which (1 chest pathology and 1 reduced GCS) are transported to King’s College Hospital, the local major trauma centre.
09:07	MMH on diversion for any patients not involved in the major incident
10:01	Mutual aid is requested by SECAmb through unofficial channels.from London Ambulance Service. London ambulance service informed at 10:45 that they had no vehicles available to support SECAmb due to significant pressures.
10:20	Tactical advisor to Silver officer 1 arrives at Coxsheath EOC
10:30	Silver officer 2 arrives on scene and takes over role of AIC Silver officer 1 arrives at Coxsheath EOC
10:52	East of England Ambulance Services are able to provide support and dispatch 5 vehicles.
10:56	MMH outpatient and elective services are shut down
10:56	NHS England receive a report from SECAmb of 10–20 casualties with additional 75 walking wounded.
10:58	NHS England declares a Major Incident
11:05	Last patient evacuated from scene
11:20	Last patient arrives at hospital
11:48	NHS England holds first meeting
13:45	Major incident stand down declared by SECAmb
14:00	SCG organised by police
15:00	Major incident stand down declared by MMH
16:26	Major incident stand down declared by NHS England
17:00	Major incident stand down declared by Kent police
17:30	Sheppey Crossing Bridge Re-opened

#### On-scene EMS work

There were hundreds of people milling around on the bridge which, in addition to safety issues, made it difficult to assess and triage casualties. The first AIC liased with senior counterparts from the police and fire services and asked them to clear everyone that was still walking, off the bridge and to a Casualty Clearing Station (CCS). A practitioner paramedic (PP) was assigned to triage the walking wounded at the CCS while ambulance crews were sent in teams of 3 to triage the casualties who remained on the bridge. Each team was assigned a car, each of which by this point had been numbered by the police. They were to be involved in extrication and stabilisation on scene if necessary and then transport the casualty to an awaiting ambulance. An ambulance parking officer was assigned to control the ingress and egress of ambulances.

#### Triage

The Sieve and sort method of triage [2] was used for casualties on site and tagging was carried out using cruciform cards and wrist slappers. Although SECAmb crews were ordered to stabilise casualties before transport, there were no reports of invasive procedures or interventions. Due to a lack of a loggist on scene during triage and recovery, there is a paucity of official information on this.

Two Hazardous Area Response Teams (HART) were deployed to the incident and assisted with triage. It was felt by both the first Ambulance Incident Commander (AIC) and the designated AIC who arrived later that a medical incident officer (MIO) or Medical Emergency Response Improvement Team (MERIT) were not needed. The triage and transport of patients was under control and their aim was to get the injured away form the scene and to hospital as soon as possible.

#### Medical communication difficulties

Prior to the arrival of the silver manager on scene, senior command was finding it difficult to obtain any hard information about the incident. It wasn’t until 11:00, when the communications officer had been set up and the last casualty had been transported from scene (11:05) that accurate information started to come through.

The NHS commissioning board recommends the formation of a Strategic Coordinating Group (SCG) for large or widespread incidents that require multi- agency discussion and coordination [3]. This is usually formed and chaired by the police but the police did not hold a SCG meeting until 14:00. This meant that there was no designated meeting point for all the agency tactical and strategic levels to assemble and coordinate a formal command and control response.

#### Structure of medical incident command during the major incident

The NHS commissioning board has published a framework for command and control structure in response to a major incident [4]. It follows the nationally recognised structure of bronze (operational), silver (tactical) and gold (strategic) command [5].

The bronze manager is normally the first level of command to be established. In this case, it was the first paramedic of enough seniority on-scene who established herself as the temporary AIC. A number of other bronze managers were allocated specific roles on-scene following this (eg. casualty clearing officer, communications officer). The silver manager develops a tactical plan to achieve the objective set out by the gold command. In this case, there were 2 silver managers: one on-scene who took over the role of AIC from the first bronze manager on scene and one off-scene who established himself at one of the EOCs. The gold manager has overall control of the organisation and liases with gold command of other task forces.

#### On-scene resources

Over 200 members of the public were involved in this incident. It was attended by 24–30 policemen (12–15 police vehicles) and 31 firemen (6 fire vehicles). There were approximately 20 ambulance crew members and in addition to this, 5 paramedic practitioners (PPs) and 3 critical care paramedics (CCPs). One air ambulance crew and 2 HART crews were also deployed. The council and various charity organisations provided food and water.

#### Hospitals receiving patients

37 casualties were taken to receiving hospitals by the emergency services. A further 32 casualties attended one of two local minor injuries units. The receiving hospitals, the relative distribution of patients, the distance from the incident scene and the type of Hospital are detailed in Table 2.

**Table 2:** Hospitals receiving casualties from the Sheppey crash

Hospital	Patient numbers	Distance from scene (Km in airline)	Type of hospital
Kings college	2	58	Major trauma centre
Kent & Canterbury	2	28	Regional hospital
Medway	14	13	Trauma unit
William Harvey	11	29	Trauma unit
QEQM	4	46	Regional hospital
Maidstone	4	21	Regional hospital
Sittingbourne memorial hospital	6	6	Minor injuries unit
Sheppey community hospital	26	4	Minor Injuries unit
Total	69		

Casualties arriving at the Medway Maritime Hospital (MMH) were triaged by the Emergency department registrar into either the resuscitation area or the trolley area. They were not given a formal category (expectant, immediate, observation, wait and dismiss) as advised in the hospital’s major incident plan. At the William Harvey Hospital, casualties were triaged on arrival by the nurse looking after them into 4 categories (immediate, urgent, delayed, fatality) according to the hospital’s major incident plan. There is no information on the triage system used in the other receiving hospitals.

The ambulance service is responsible for casualty distribution and alerting the receiving hospital(s). Casualty distribution is a dynamic decision based on injuries, capacity, access and egress.

### Patient characteristics

There were no deaths reported from this incident. Due to data sharing restrictions, only details of the 25 casualties attending the Medway maritime Hospital and the William Harvey Hospital could be obtained. Of these patients:

- □ Male: female ratio was 13:12
- □ Age range was 19–66 years old
- □ Twenty patients were discharged home the same day, 4 patients were discharged the following day and 1 patient was discharged 6 days later
- □ One patient was admitted to the High Dependancy Unit (HDU) for monitoring overnight but was discharged the next morning
- □ Six patients sustained fractures (2 scaphoid, 2 tibia/fibula, 1 clavicle, 1 sternal, 1 distal radius, 1 great toe)
- □ The majority of injuries were soft tissue injuries requiring no follow up

### Key lessons

#### Problems

- □ Late declaration of a major incident across agencies
- □ Poor communication to and amongst senior command in the early, crucial phase of the response, both within and between agencies
- □ Lack of familiarity of members of staff with major incident protocols
- □ Late involvement of Senior command for Ambulance, Police and Fire.
- □ Late arrival of SECAmb snior managers at their respective posts due to distance needed to travel, traffic conditions and late decisions on command structure
- □ Delayed set up of the SCG

#### Successes

- □ All casualties were cleared from the scene in under 4 h due to coordinated efforts by ambulance, police and fire services on-scene
- □ Effective & Efficient triage of casualties by highly skilled paramedics in the form of CCPs who could triage and manage the severely injured patients and PPs who could triage and eyeball the walking wounded and walking well
- □ A major incident was prevented from becoming a disaster: food, water and shelter were offered to those left behind on scene after the last patient was taken to hospital. Voluntary organisations and the local council were central to facilitating this

## Consent

No consent required.

## Abbreviations

AIC: Ambulance Incident Commander
CCP: Critical Care Paramedic
CCS: Casualty Clearing Station
EMS: Emergency medical Services
EOC: Emergency Operations Centre
EPRR: Emergency Preparedness Resilience and Response
HART: Hazardous Area Response Team
HDU: High Dependancy Unit
MIO: Medical Incident Officer
MERIT: Medical Emergency Response Improvement Team
MMH: Medway maritime Hospital
NHS: National Health Service
PP: Paramedic Practitioner
P1: Priority 1
P2: Priority 2
P3: Priority 3
SCG: Strategic Coordination Group
SECAmb: South East Coast Ambulance
